# Age and sex: dual drivers remodeling the anti-tumor immune microenvironment and shaping personalized immuno-oncology

**DOI:** 10.3389/fimmu.2026.1918543

**Published:** 2026-09-01

**Authors:** Yipeng Wang, Tianshu Wang, Ying Xi, Yihan Wang, Yibo Bai, Tao Zhang

**Affiliations:** 1 Department of Gastric Surgery, Cancer Hospital of Dalian University of Technology, Liaoning Cancer Hospital & Institute, Shenyang, Liaoning, China; 2 Medical Oncology Department of Gastrointestinal Tumors, Cancer Hospital of Dalian University of Technology, Liaoning Cancer Hospital & Institute, Shenyang, Liaoning, China; 3 Department of Laboratory Medicine, Shengjing Hospital of China Medical University, Shenyang, Liaoning, China; 4 Liaoning Clinical Research Center for Laboratory Medicine, Shenyang, Liaoning, China; 5 China Medical University, Shenyang, Liaoning, China

**Keywords:** anti-tumor immunity, immune evasion, immunosenescence, sex differences, tumor microenvironment

## Abstract

Despite breakthrough advancements in cancer immunotherapy, significant inter-individual heterogeneity in clinical outcomes persists, bringing the regulatory roles of intrinsic host biological variables into sharp focus. Accumulating fundamental and clinical evidence indicates that age and sex play crucial roles in determining tumor susceptibility, disease progression, and the remodeling of the anti-tumor immune microenvironment. This review systematically delineates the profound impacts of the dual dimensions of age and sex on anti-tumor immune responses and immune evasion mechanisms. In the dimension of age, this article outlines the progressive functional decline of T/B lymphocytes and innate immune subsets driven by immunosenescence, and emphatically reveals how inflammaging and its associated senescence-associated secretory phenotype (SASP) orchestrate the formation of an immunosuppressive tumor microenvironment. In the dimension of sex, we deeply explore four core mechanisms comprising sex chromosome genomics (e.g., escape from X-chromosome inactivation and loss of Y chromosome), sex hormone networks, microenvironmental metabolic reprogramming, and the host gut microbiome, elucidating the molecular basis driving the disparities in innate and adaptive immunity between males and females. In summary, thoroughly deciphering the complex immune regulatory networks driven by age and sex not only helps elucidate the disparities in efficacy and toxicity observed in patients undergoing immune checkpoint inhibitors, but also provides crucial theoretical foundations and translational insights for the future development of “age-tailored” and “sex-specific” strategies in personalized immuno-oncology.

## 1 Introduction

In recent years, cancer immunotherapy, predominantly represented by immune checkpoint inhibitors (ICIs), has revolutionized the clinical treatment landscape for advanced malignancies. However, substantial clinical practice and epidemiological data indicate profound inter-individual heterogeneity in patient response rates and survival benefits following immunotherapy (1, 2). Previous studies have conventionally attributed these disparities to the tumor mutational burden (TMB) or external environmental exposures (i.e., modifiable factors such as smoking and dietary habits) (3, 4). Today, an increasing body of scientific evidence suggests that fundamental systemic biological variables of the host (though certain endocrine characteristics can be clinically adjusted), which have historically been underrecognized, collaborate with previously known factors such as TMB to modulate the tumor microenvironment (TME) and therapy responses (5).

Among the multitude of intrinsic host factors, age and physiological sex stand out as the two most fundamental and critical biological dimensions. They not only profoundly shape the basal developmental trajectories of the innate and adaptive immune systems but also play a dominant role in tumorigenesis, progression, and immune evasion (6–8). In light of this, the present review systematically explores the profound impacts of age and sex on anti-tumor immune responses. First, we will elucidate how immunosenescence and inflammaging synergistically orchestrate an immunosuppressive microenvironment in the aging host. Subsequently, we will deeply dissect the immune evasion mechanisms mediated by sexual dimorphism across four core dimensions: sex chromosome genomics, sex hormone networks, metabolic reprogramming, and the host microbiome. By comprehensively decoding these two core variables, this review aims to provide a crucial theoretical foundation for the future development of “age-tailored” and “sex-specific” strategies in personalized immuno-oncology.

## 2 Age-associated immune remodeling: immunosenescence and tumor immune evasion

Host age represents a core non-modifiable biological variable dictating tumorigenesis, disease progression, and immunotherapy efficacy. Epidemiological data consistently indicate that the vast majority of malignancies are fundamentally age-related diseases, with their incidence risk rising exponentially with advancing age. The underlying biological root of this age-dependent tumor susceptibility lies in the progressive and irreversible structural remodeling and functional decline of the host immune system over time—a phenomenon termed “immunosenescence” (9, 10).

Concurrently, the aging host is frequently accompanied by a systemic, low-grade, and persistent state of chronic sterile inflammation, termed “inflammaging” (11, 12). During tumor evolution, the functional “degeneration” (senescence) and abnormal “overactivation” (inflammation) of the immune system intertwine and act synergistically within the TME. This mutually reinforcing cycle not only severely impairs the host’s early immune surveillance against malignant mutant cells but also provides a highly fertile ground for tumor immune evasion through the recruitment of complex immunosuppressive cellular networks. The following sections will systematically dissect the core characteristics of both immunosenescence and inflammaging, and explore the profound impacts of age on the tumor immune microenvironment and clinical immunotherapy responses. Although this review centers primarily on immunosenescence and inflammaging in elderly populations, it is necessary to recognize the opposite end of the age continuum, namely oncology in pediatric patients and young adults. To maintain a cohesive focus on aging-associated mechanisms, the distinct immune microenvironments and characteristic tumor biology observed in these younger cohorts fall outside our primary scope. These topics merit independent, specialized exploration in future dedicated reviews and empirical studies.

### 2.1 Core characteristics of immunosenescence and their association with anti-tumor immunity

Aging is widely recognized as a major risk factor for the vast majority of known malignancies. A hallmark of the aging process is the structural remodeling and functional imbalance of the immune system, which profoundly affects both innate and adaptive immunity. Within the innate immune compartment, functional defects primarily manifest as reduced neutrophil production, diminished phagocytic and chemotactic capacities, and an increased susceptibility to apoptosis (13).

Although the senescence of the innate immune system is generally considered less pronounced than that of adaptive immunity, it exerts an equally decisive impact on anti-tumor immune outcomes. As the most potent professional antigen-presenting cells, dendritic cells (DCs) are responsible for the uptake, processing, and presentation of tumor antigens, serving as the central hub for initiating adaptive immune responses (14). However, in elderly individuals, DCs experience a significant decline in crucial functions; they exhibit not only a numerical decline but also impaired capacities for antigen uptake, endocytosis, and interferon production (15). Furthermore, the downregulated expression of co-stimulatory molecules (e.g., CD80, CD86) renders DCs incapable of effectively priming naive T cells, ultimately leading to a severe blockade in the initiation of anti-tumor immunity (14).

Natural killer (NK) cells, the core effector cells of innate immunity, can directly eradicate tumor cells without prior antigen sensitization. With advancing age, impaired granule exocytosis and defective activation-associated signaling pathways lead to a significant decline in the cytotoxicity and cytokine secretion of NK cells (16). Moreover, senescent NK cells exhibit a comprehensive downregulation of natural cytotoxicity receptors (NCRs), such as NKp30, NKp46, and NKp44, which significantly diminishes their ability to recognize and kill tumor cells. Concurrently, the compromised ability of senescent NK cells to produce key cytokines (e.g., IFN-γ) and chemokines further undermines the host’s early line of defense against tumors and exogenous infections (17).

Macrophages, as crucial resident immune cells within the tumor microenvironment (TME), are similarly profoundly affected by aging. Driven by the continuous accumulation of senescence-associated cytokines and the substantial enrichment of local inflammatory mediators in the TME, macrophages show marked upregulation of pro-inflammatory gene expression and are highly prone to polarize toward a pro-tumorigenic tumor-associated macrophage (TAM) phenotype. These senescent, polarized macrophages, accompanied by severely compromised capacities for cellular proliferation and DNA damage repair, secrete a unique array of tumor-promoting SASP factors—including BMP2, IL-10, CCL2, CCL7, CCL8, and CXCL13—thereby forging a robust immunosuppressive barrier that ultimately accelerates tumor proliferation and progression (18, 19).

However, the senescence of the adaptive immune system is often even more pronounced, rendering it the major factor of anti-tumor immune deficiency in the elderly. Within the adaptive immune system, T-cell senescence constitutes the core component of immunosenescence. Extensive studies in murine models have confirmed that T lymphocytes undergo multiple phenotypic alterations and progressive functional decline with aging. Continuous thymic involution post-puberty leads to a shrinkage of the naive T-cell pool, decreased T-cell receptor (TCR) diversity, and an imbalance in memory T-cell subsets, ultimately impairing the host’s antigen recognition capacity (13). These alterations significantly elevate the risk of autoimmune diseases, chronic autoinflammation, infections, and malignancies in the elderly. With advancing age, T cells downregulate the expression of the CD28 molecule, resulting in the emergence of CD4^+^CD28⁻ and CD8^+^CD28⁻ T-cell subsets. This represents a hallmark of replicative senescence and is closely associated with impaired vaccine responsiveness (20, 21). The functional exhaustion and numerical decline of tumor antigen-specific CD8^+^ T cells are critical indicators of accelerated tumor growth and immunotherapy resistance in elderly individuals. Notably, the adoptive transfer of antigen-specific T cells from young mice can effectively suppress tumor expansion and restore sensitivity to PD-1 blockade therapy in aged mice (20).

In the aging host, senescent T cells are characterized not only by reduced cytokine secretion but also by upregulated expression of inhibitory receptors (e.g., PD-1, CTLA-4, LAG-3) and immunosuppressive enzymes (e.g., CD39). Notably, while both senescent and exhausted T cells share overlapping phenotypic markers such as upregulated PD-1 and CTLA-4, it is critical to distinguish between these two states. Senescence is an irreversible cell cycle arrest driven by intrinsic aging (e.g., telomere attrition) that is generally refractory to immune checkpoint inhibitors (ICIs). In contrast, exhaustion is an epigenetically distinct, antigen-driven dysfunction within the TME that can often be reinvigorated by ICI therapies. In elderly patients, these two states frequently overlap, severely compounding immunotherapy resistance (22). Concurrently, these cells acquire a senescence-associated secretory phenotype (SASP), releasing soluble factors that broadly recruit myeloid-derived suppressor cells (MDSCs) and regulatory T cells (Tregs). This cascade fosters an immunosuppressive microenvironment that profoundly hinders effective anti-tumor immune responses (23). Furthermore, studies indicate that senescent naive T cells exhibit activation initiation defects due to CD45 upregulation, which elevates the TCR activation threshold and restricts effector cell differentiation. Finally, aging impairs mitochondrial metabolism (particularly fatty acid oxidation) and precipitates the collapse of proteostasis (including defective autophagy and exacerbated endoplasmic reticulum stress), both of which serve as fundamental molecular mechanisms driving T-cell functional deterioration (24).

Although research on immunosenescence has predominantly focused on T cells, mounting evidence indicates that B cells also undergo significant phenotypic remodeling and functional decline during aging and tumorigenesis. During the aging process, the homeostasis of naive B cells is severely compromised, likely due to a decline in the levels of BAFF/BLyS, a critical survival factor for the B-cell lineage. Conversely, memory B cells continuously accumulate with age. These cells are frequently directed against environmental and/or auto-antigens and exhibit a markedly restricted diversity within their B-cell receptor (BCR) repertoire. Mechanistic studies have revealed that the diminished induction of E47, a key transcription factor for B cells, during the activation of aged B cells leads to the insufficient expression of activation-induced cytidine deaminase (AID)—the central enzyme responsible for class switch recombination and somatic hypermutation. This defect directly results in impaired antibody affinity maturation and weakened antibody-mediated anti-tumor immune protection in the elderly (25).

To systematically elucidate the multidimensional evolution of both the innate and adaptive immune systems during the aging process, we have structurally summarized the age-related phenotypic alterations of major immune cell subsets and their specific mechanisms in undermining anti-tumor immunity (see **Table 1**).

**Table 1:** Phenotypic remodeling of immune cell subsets associated with immunosenescence and their impact on anti-tumor immunity.

Immune system	Cell subsets	Age-related functional/phenotypic changes	Effects on anti-tumor immunity	References
Inherent immunity	Neutrophile granulocyte	Decreased quantity; diminished chemotactic ability; increased susceptibility to apoptosis.	The body inherent ability to kill and eliminate tumor cells diminished, increasing the risks of infection and tumor progress.	(13)
Innate immunity	Natural killer (NK) cells	There is reduced secretion of cytokines, decreased cytotoxic activity against target cells; diminished expression of natural cytotoxic receptors; and reduced secretion of effector molecules.	The direct antigen-free sensitized cytotoxic capacity against tumor cells is diminished, rendering it incapable of effectively initiating subsequent adaptive antitumor immune responses.	(16, 17)
Innate immunity	Dendritic cells (DCs)	Decreased quantity; impaired antigen uptake and presentation capacity; reduced expression of co-stimulatory molecules; diminished interferon secretion	Failure to effectively activate initial T cells; impaired initiation of antitumor immune responses.	(14, 15)
Innate immunity	Macrophage	Expression of pro-inflammatory genes was significantly upregulated; cell proliferation and DNA damage repair capacity were impaired; polarization toward tumor-associated macrophages (TAMs)occurred; and SASP factors were secreted.	It establishes an immunosuppressive tumor microenvironment; directly promotes tumor proliferation, invasion, and metastasis; and mediates resistance to immunotherapy.	(18, 19)
Adaptive immunity	T lymphocytes (core)	Reduction in the initial T cell repertoire; decreased diversity of T cell receptors (TCRs); imbalance in memory T cell subsets; impaired replication capacity; reduced cytokine secretion; elevated expression of inhibitory receptors and immunosuppressive enzymes; mitochondrial metabolic dysfunction and protein homeostasis disruption; defective initiation of initial T cell activation.	Impaired antigen recognition capability; reduced vaccine-induced immune response; recruitment of myeloid-derived suppressor cells (MDSCs) and regulatory T cells (Tregs), leading to the formation of an immunosuppressive microenvironment.	(13, 20, 21, 23, 24)
Adaptive immunity	B leukomonocyte	Accumulation of memory B cells; limited diversity of the B-cell receptor repertoire; decreased antibody affinity leading to increased pro-inflammatory B cells.	The antibody-mediated antitumor immune protective capacity is diminished, leading to the formation of an immunosuppressive microenvironment.	(25)

### 2.2 Inflammaging: regulation of tumor immunity by age-associated chronic inflammation

Inflammaging refers to a chronic, low-grade, and systemic state of sterile inflammation that emerges during the aging process in the absence of overt exogenous infectious stimuli. The continuous elevation in the synthesis and circulating levels of inflammatory mediators severely impairs adaptive immune functions, thereby inducing and exacerbating immunosenescence. Conversely, immunosenescence further fuels the spread of inflammation, forging a self-amplifying loop. This synergistic crosstalk significantly propels the onset and progression of various age-related pathologies, including infections, malignancies, chronic inflammatory diseases, and neurodegenerative disorders (26).

The underlying mechanisms driving inflammaging are highly complex and primarily encompass the following core dimensions. The first is the continuous accumulation of senescent cells. Although these cells have permanently exited the cell cycle (irreversible growth arrest), they persistently secrete a plethora of highly active biomolecules, including pro-inflammatory cytokines, chemokines, proteases, and growth factors—a hallmark feature termed the senescence-associated secretory phenotype (SASP). While SASP secretions may transiently activate the immune system to clear damaged cells under specific physiological contexts, they predominantly exert potent pro-tumorigenic effects within the tumor microenvironment. SASP not only directly drives the malignant proliferation of pre-malignant and tumor cells but also induces epithelial-mesenchymal transition (EMT), ultimately accelerating tumor invasion and distant metastasis (27, 28).

Second is gut dysbiosis. The human gut microbiota undergoes chronological evolution and structural remodeling with advancing age: overall microbial richness declines significantly, accompanied by a profound shift in community attributes—beneficial commensals (e.g., Clostridiales, Bifidobacterium) gradually diminish, whereas the proportion of opportunistic pathogens (e.g., Enterobacteriaceae) continuously rises. The gut microbiota interacts intricately with the host through multiple avenues, including the release of diverse metabolites, mediation of epigenetic regulation, and activation of non-canonical signaling pathways. This age-related microbial dysbiosis aberrantly activates the host’s innate immune system, thereby inducing and driving a myriad of aging-associated pathologies (29).

Third is mitochondrial dysfunction. Because the mitochondrial interior constitutes a highly oxidative microenvironment and mitochondrial DNA (mtDNA) lacks histone protection, its damage repair efficiency is significantly lower than that of nuclear DNA, rendering it highly susceptible to genetic mutations and structural damage (30). As the central hubs of cellular energy metabolism, mitochondria are also deeply involved in intracellular signal transduction, reactive oxygen species (ROS) metabolism, cellular homeostasis regulation, and apoptotic processes. Concomitant with the aging process, mitochondria undergo profound alterations in number, morphology, and physiological function, typically manifesting as decreased mitochondrial membrane potential, insufficient adenosine triphosphate (ATP) synthesis, impaired mitophagy, and the leakage and accumulation of damaged mtDNA into the cytoplasm. The underlying triggers encompass the suppression of the mTOR/TFEB pathway, downregulated expression of mitochondrial 8-oxoguanine DNA glycosylase (mtOGG1), and the blockade of the PINK1/Parkin-mediated ubiquitination-degradation pathway. The aforementioned alterations collectively result in the accumulation of damaged mitochondria, which further exponentially amplifies the generation of reactive oxygen species (ROS) and potently drives the SASP-related inflammatory cascade (31). Notably, growth differentiation factor 15 (GDF-15) serves as a critical biomarker reflecting mitochondrial dysfunction. Studies have demonstrated that its expression levels can surge up to 200-fold above normal physiological baselines in various malignancies, including melanoma, breast, colorectal, pancreatic, cervical, and prostate cancers (32).

Inflammaging impairs anti-tumor immune responses through multiple pathways, thereby fueling tumorigenesis and progression. First, inflammaging triggers the massive systemic release of inflammatory mediators, primarily encompassing tumor necrosis factor-α (TNF-α), C-reactive protein (CRP), and a diverse array of interleukins (e.g., IL-6, IL-1 receptor antagonist [IL-1ra], IL-18, and IL-1β). This chronic inflammatory milieu severely compromises the capacity of the adaptive immune system to resolve inflammation. For instance, studies in breast cancer have revealed that elevated IL-6 not only significantly promotes tumor progression but also highly correlates with advanced age and poor patient prognosis (33, 34). The persistent exacerbation of inflammation and the excessive accumulation of cytokines can aberrantly dysregulate the cell cycle and proliferative potential of immune cells, ultimately mediating the onset of immunosenescence and diminishing the body’s overall anti-tumor immunity (35). Second, an overload of these active substances—including inflammatory mediators, growth factors, cytokines, and chemokines—can directly stimulate tumor angiogenesis, mediate tumor cell invasion and metastasis, and induce multidrug resistance. More lethally, they broadly recruit immunosuppressive subsets, such as regulatory T cells (Tregs) and myeloid-derived suppressor cells (MDSCs), thereby completely remodeling the tumor microenvironment into a profoundly immunosuppressive state (36, 37).

It is worth noting that inflammaging is not an irreversible, absolute pathological process. Beyond chronological age, lifestyle interventions can effectively modulate inflammaging (38). For instance, regular resistance training and multicomponent exercise interventions can mitigate chronic low-grade inflammation by regulating multiple inflammatory pathways. Among current dietary patterns, the Mediterranean diet (rich in polyphenols, unsaturated fatty acids, and dietary fiber) is recognized as one of the most effective anti-inflammatory diets due to the synergistic effects of its diverse nutritional components. Furthermore, sleep quality and psychological stress profoundly impact systemic inflammatory activity: short sleep duration and poor sleep quality exert pro-inflammatory effects; circadian rhythm disruptions lead to endocrine imbalance, affecting mitochondrial function; while chronic psychological stress persistently activates the hypothalamic-pituitary-adrenal (HPA) axis, inducing a pro-inflammatory physiological state (38, 39).

At the intersection of endocrinology and metabolism, premenopausal 17β-estradiol primarily exerts anti-inflammatory effects, whereas postmenopausal estrone (synthesized mainly in adipocytes) promotes NF-κB-driven inflammatory responses. Because obesity-related chronic inflammation can accelerate the functional decline of immune cells, strict weight management and metabolic interventions may serve as effective adjunctive strategies to improve the anti-tumor immune microenvironment (40). Meanwhile, adequate vitamin D supplementation can positively impact overall health in menopausal women by regulating immune function and inhibiting tumor cell proliferation (41). Therefore, incorporating lifestyle and metabolic assessments is of significant importance when formulating personalized tumor immunotherapy regimens.

### 2.3 The impact of age on the tumor immune microenvironment and immunotherapy efficacy

The tumor microenvironment (TME) is a highly complex and dynamic ecological system intricately composed of various immune cells, stromal cells, vascular endothelial cells, adipocytes, extracellular matrix (ECM), and soluble factors. During tumor evolution, the TME not only promotes tumor proliferation and invasion but also systematically regulates the host immune status and mediates therapeutic resistance (42).

Advancing age significantly remodels the immunological landscape of the TME, causing the microenvironment in elderly patients to exhibit a profoundly classical immunosuppressive phenotype. On the one hand, the intratumoral infiltration of anti-tumor effector cells (e.g., CD8^+^ T cells, NK cells, and M1-polarized macrophages) is drastically reduced and accompanied by severe functional exhaustion; concurrently, immunosuppressive subsets (e.g., Tregs, MDSCs, and M2-polarized macrophages) are massively enriched in the local milieu (43). On the other hand, senescent stromal cells within the TME secrete an abundance of SASP-related factors—such as transforming growth factor-beta (TGF-β) and matrix metalloproteinases (MMPs)—which potently drive the aberrant remodeling and cross-linking stiffening of the ECM. This highly stiffened matrix forms a formidable physical barrier that not only severely impedes the deep infiltration of effector immune cells but also significantly restricts the effective delivery of immunotherapeutic agents into the tumor parenchyma (44). Consequently, the incidence risk and progression rates of the vast majority of malignancies rise significantly with age, a macroscopic epidemiological trend that is mechanistically inseparable from the continuous accumulation of senescent immune and stromal cells in the aging host (45, 46). Taking CD8^+^ T cells as an example, senescent CD8^+^ T cells are significantly enriched not only in healthy elderly populations but also in young individuals with chronic viral infections and patients with various malignancies. Having undergone profound phenotypic remodeling, these senescent T cells lose their inherent cytotoxic killing capacity and instead persistently exert a negative, immunosuppressive role against anti-tumor immune responses within the tumor microenvironment. More crucially, extensive clinical evidence demonstrates that across a broad spectrum of cancers—including lung cancer, gastric cancer, renal cell carcinoma, glioblastoma, non-Hodgkin lymphoma, chronic lymphocytic leukemia, and acute myeloid leukemia—the infiltration abundance of senescent CD8^+^ T cells within the tumor lesions exhibits a significant positive correlation with poor patient prognosis. That is, a higher level of their infiltration invariably dictates worse overall survival and clinical outcomes for the patients (47).

Immune checkpoint inhibitors (ICIs) have emerged as the core therapeutic modality for a variety of advanced malignancies; however, patient age exerts a particularly profound impact on the ultimate clinical outcomes of these immunotherapies. The functional deterioration of the immune system accompanying aging not only alters the therapeutic response to ICIs in elderly patients but also drives significant efficacy disparities across different age cohorts, with inter-individual heterogeneity being exceptionally pronounced among the elderly.

**Figure 1:**
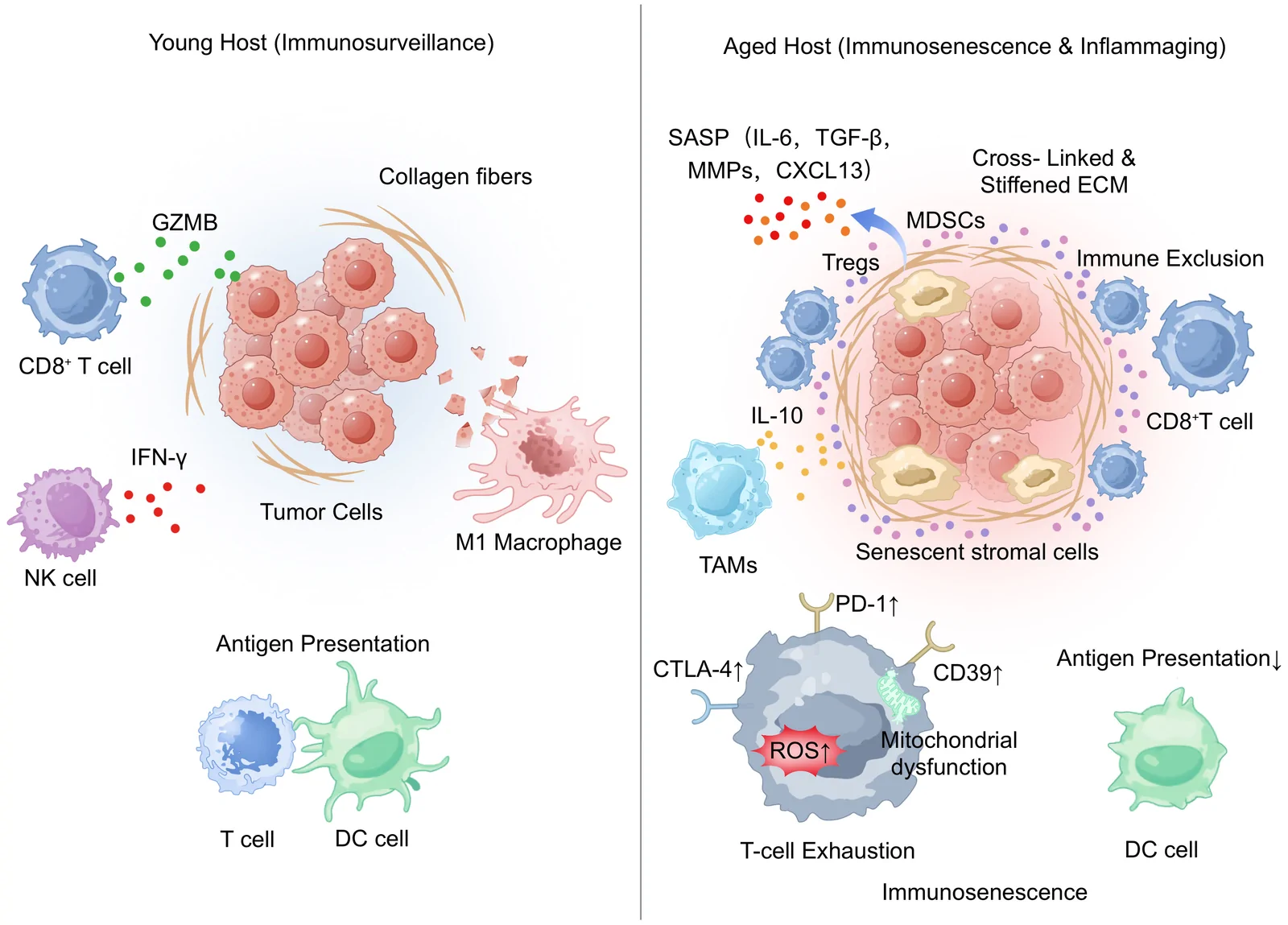
Age-driven remodeling of the tumor immune microenvironment (TME).

Although macroscopic data suggest that both elderly (≥65 years) and young cancer patients can derive survival benefits from ICI therapy, their baseline immune phenotypes differ significantly. However, it is vital to put these experimental findings into a clinical perspective. Several comprehensive retrospective analyses—most notably the study by Nebhan et al. (2021) have demonstrated that the overall immunotherapy efficacy (e.g., objective response rate and progression-free survival) in elderly and even extremely old patients (≥85 years) is often remarkably similar to that in younger cohorts. Furthermore, the incidence of severe immune-related adverse events (irAEs) does not significantly increase with age. Nevertheless, due to reduced physiological reserves, elderly patients exhibit a significantly higher rate of ICI discontinuation following irAE onset (48). This highlights the intricate complexity of age-related immune responses and underscores that advanced age alone, despite the profound TME remodeling, should not preclude patients from receiving ICI therapies, provided that personalized toxicity management is implemented. Specifically: first, elderly patients exhibit a unique peripheral immune cell composition, characterized by significantly reduced proportions of naive cytotoxic T cells (TcN), naive helper T cells (ThN), B cells, and double-negative T cells (DNT), alongside a compensatory elevation in the proportion of NK cells. Second, following ICI administration, the overall magnitude of peripheral cytokine responses is markedly blunted in elderly patients. Finally, the relative expression levels of immune checkpoint molecules (e.g., PD-1, TIGIT) on immune cells are inherently higher in the aging host (49, 50). Furthermore, subgroup analyses stratified by tumor type, line of therapy, and immunotherapy regimen have revealed a specific phenomenon: in melanoma cohorts, young patients achieve significantly superior progression-free survival (PFS) benefits following immunotherapy compared to their elderly counterparts, a discrepancy largely attributed to the profound functional decline of immune cells in the aged population (51). The multidimensional mechanisms underlying age-driven TME remodeling and immune evasion are schematically summarized in **Figure 1**.

## 3 Sex-driven immune remodeling: anti-tumor immune heterogeneity and therapeutic responses

Biological sex, as the most fundamental biological variable, leaves an indelible imprint on shaping the developmental trajectories and response patterns of the host immune system. According to the GLOBOCAN 2022 data released by the International Agency for Research on Cancer (IARC), the lifetime risk of cancer mortality in males globally (approximately 1 in 9) is significantly higher than that in females (approximately 1 in 12). Notably, this pronounced heterogeneity is not only widely prevalent in the incidence and progression of non-reproductive system tumors—such as lung, colorectal, gastric, and liver cancers—but also profoundly affects the ultimate clinical efficacy of tumor immunotherapies, particularly immune checkpoint inhibitors (52).

Historically, these sex disparities were often simplistically attributed to adverse environmental factors, such as smoking, alcohol consumption, and occupational exposures. However, even after statistically adjusting for these modifiable variables, the survival and immune response differences conferred by sex remain robustly significant (53–55). This consistently indicate suggests that the intrinsic biological characteristics of the host act as the core driving force behind this phenomenon. Accumulating research confirms that males and females possess an inherent “sexual dimorphism” at the baseline of both innate and adaptive immunity (56, 57). This divergence in immune phenotypes does not exist in isolation; rather, it is the profound biological consequence jointly driven by multidimensional factors: the innate imprint of sex chromosome genomics, the dynamic regulation of sex hormone endocrine networks, systemic metabolic reprogramming, and the distal mediation of the host gut microbiome (58–60). The following sections will deeply dissect these four core dimensions to elucidate how sex disparities remodel the anti-tumor immune microenvironment and precipitate tumor immune evasion.

### 3.1 Impact of sex chromosomes and genomic disparities on anti-tumor immunity

The X and Y sex chromosomes encode the most fundamental genetic differences between males and females. Typical male (XY) and female (XX) karyotypes exhibit fundamental divergences in their gene expression patterns. Specifically, although female cells inherit one X chromosome from each parent, to maintain gene dosage balance between the sexes, they initiate an epigenetic silencing mechanism during early embryonic development. This process highly condenses and transcriptionally silences one X chromosome copy, rendering it the inactive X chromosome (Xi)—a phenomenon known as X-chromosome inactivation (XCI) (61, 62). However, this transcriptional silencing mechanism is not absolute. Extensive studies have revealed that the X chromosome is highly enriched with a multitude of crucial immune-regulatory genes, and a subset of these genes can undergo “escape from XCI.” It is precisely this escape phenomenon, driven by differential sex chromosome expression, that directly mediates the pronounced heterogeneity between males and females at the baseline of both innate and adaptive immunity, playing a critical role in remodeling anti-tumor immune responses (63, 64).

Toll-like receptor 7 (TLR7) serves as the quintessential archetype elucidating this mechanism. As a critical member of the TLR family and a type I transmembrane glycoprotein, the TLR7 gene is definitively and exclusively located on the X chromosome. Its expression exhibits marked cellular specificity, being predominantly highly expressed in plasmacytoid dendritic cells (pDCs), macrophages, B cells, and specific T-cell subsets. Notably, pDCs act as the primary carriers of TLR7; upon activation, they secrete massive amounts of tumor necrosis factor (TNF) and type I interferons (IFN-I), thereby potently initiating the innate immune response (65, 66).

More crucially, TLR7 functions not only as a “radar” for innate immunity but also as a bridge to adaptive immunity. The robust IFN-I production triggered by its activation significantly promotes the maturation of dendritic cells, upregulating the expression of surface co-stimulatory molecules (CD80 and CD86). This, in turn, enhances their antigen-presenting capacity and bolsters the cytotoxic functions of tumor-specific T cells (67, 68). Concurrently, TLR7 signaling can directly act on B cells, fostering their activation, proliferation, and the induction of specific antibodies (69).

It is imperative to emphasize that, precisely due to the escape from X-chromosome inactivation, TLR7 achieves biallelic transcription and expression in female immune cells. Compared to the monoallelic expression observed in males, this gene dosage doubling effect empowers females to trigger the aforementioned immune cascades more rapidly and exponentially upon encountering tumor-associated antigens. This gene-dosage advantage not only endows females with a more robust early anti-tumor immune response but also effectively counteracts and curbs tumor immune evasion during the nascent stages of tumorigenesis (70). Another classic paradigm elucidating immune sexual dimorphism is the KDM6A (also known as UTX) gene, which is similarly located on the X chromosome. It has been validated as one of the most stable genes undergoing “escape from X-chromosome inactivation,” primarily encoding a histone H3K27 demethylase. KDM6A epigenetically regulates immune-related gene expression predominantly through two distinct mechanisms. In the catalysis-dependent mechanism, the UTX protein directly activates gene expression by mediating proximal H3K4 monomethylation and H3K27 acetylation. In the catalysis-independent mechanism, UTX recruits the MLL3/4 complex to the promoters or enhancers of target genes, where MLL3/4 mediates H3K4 monomethylation to facilitate the transcription process (71).

Within the immune microenvironment, KDM6A fine-tunes the transcriptional balance between pro-inflammatory and anti-inflammatory genes, thereby profoundly influencing the differentiation, polarization, and activation states of key immune subsets, including monocytes, macrophages, dendritic cells, and helper T cells (72). Particularly in the context of anti-tumor immunity, KDM6A drives robust anti-tumor immune responses and restricts tumor growth by binding to and regulating genes involved in immune recognition and cytokine signaling. Specifically, KDM6A binds to and activates the enhancer regions of NLRC5 and CIITA—master transcriptional regulators of the major histocompatibility complex (MHC)—thereby significantly upregulating the expression of MHC class I and II molecules. This epigenetic mechanism effectively maintains tumor immunogenicity and prevents tumor cells from undergoing immune evasion.Furthermore, the sustained expression of a multitude of critical immune-related cytokines and chemokines—including T-cell chemoattractants (CCL5 and CCL7), inflammatory chemokines (CCL3), interferon-alpha 2 (IFN-α2), and Fms-related receptor tyrosine kinase 3 ligand (FLT3L)—is highly dependent on the presence of KDM6A. Studies in murine models have demonstrated that the genetic ablation of KDM6A leads to a devastating remodeling of the tumor immune microenvironment, characterized by a drastic reduction in the intratumoral infiltration of various immune cells, encompassing CD4^+^ T cells, CD8^+^ T cells, regulatory T cells, NK cells, granulocytes, and monocytes (73). Under cellular stress conditions, the UTX protein can also exert catalysis-independent anti-tumor effects: via its TPR domain, it competitively binds to the G3BP1 protein, thereby disrupting the scaffold network structure of stress granules (SGs) and triggering their disassembly. This alternative mechanism similarly suppresses tumor progression (74).

Taken together, courtesy of the escape from X-chromosome inactivation, the KDM6A gene stably maintains two functional expression copies (biallelic expression) in females. Compared to males, this innate advantage in gene dosage provides females with a uniquely superior protective barrier for anti-tumor immunity (75). This is robustly corroborated by both clinical and fundamental evidence: within the male tumor microenvironment, T cells are exceptionally prone to functional exhaustion, thereby accelerating tumor deterioration; conversely, in females, T cells are more inclined to sustain prolonged, high-level effector functions, thereby more effectively curbing tumor progression (76).

In stark contrast to the innate advantages conferred by the female X chromosome, the phenomenon of Loss of Y chromosome (LOY) is significantly associated with tumorigenesis, progression, and poor prognosis in males. This pathogenic process is primarily attributed to the deletion of critical tumor suppressor genes located on the Y chromosome, such as TMSB4Y, KDM5D, and UTY. Specifically, the mutation or loss of TMSB4Y not only enhances tumor cell proliferation but also induces alterations in cell membrane composition, thereby facilitating tumor immune evasion. The KDM5D gene encodes a histone demethylase; serving as a core epigenetic regulator, it fine-tunes the expression of various immune-related genes via demethylation modifications (77). Studies have demonstrated that the loss of KDM5D aberrantly elevates H3K4me3 levels at the promoter regions of target genes, thereby significantly amplifying tumor invasiveness (78). In terms of remodeling the TME, LOY-bearing tumors potently promote the functional exhaustion of local CD8^+^ T cells. This phenomenon is largely driven by LOY-induced massive enrichment of immunosuppressive PD-L1-positive macrophages, which triggers T-cell-mediated immune evasion and the ensuing exhaustion of CD8^+^ T cells. Mechanistically, this immunosuppressive cascade is closely linked to the deficiency of UTY (the Y-chromosome homolog of KDM6A, albeit with minimal demethylase activity) or KDM5D (77, 78). Furthermore, LOY occurs not only in malignant tumor cells but is also highly prevalent in immune cells within the peripheral blood and the TME. A model proposed by Chen et al. profoundly elucidates the link between immune cell LOY and tumor progression: the occurrence of LOY in T cells within the TME leads to an aberrant functional enhancement of regulatory T cells (Tregs), thereby further magnifying local immunosuppressive effects. Simultaneously, CD8^+^ T cells harboring LOY exhibit a marked decline in activation levels, TCR signal transduction efficiency, and cytotoxic signatures, resulting in a significantly diminished capacity for direct tumor killing. Evidently, the phenomenon of LOY not only triggers massive chromosomal aberrations and the malignant evolution of tumor cells themselves but also induces a comprehensive exhaustion of the host’s anti-tumor immune functions at the microenvironmental level. These two pathogenic events forge a self-amplifying loop within male patients, profoundly accelerating tumor progression (79). In summary, by systematically suppressing the functions of effector immune cells and synergistically downregulating the inherent immunogenicity of tumor cells, the loss of Y chromosome (LOY) ultimately serves as a core genomic event driving tumor immune evasion in males.

### 3.2 Dynamic regulation by sex hormone networks and microenvironmental immune evasion

Androgens exert broad immunosuppressive effects on the host immune system primarily through the androgen receptor (AR) signaling pathway. Given that the AR is expressed across virtually all immune cell subsets, its activation concomitantly and profoundly impacts both innate and adaptive immune responses. Within the adaptive immune compartment, CD8^+^ T cells endogenously express robust levels of the AR (80). Mechanistic studies reveal that the hyperactivation of AR not only directly downregulates the expression of critical genes essential for the cytotoxic and effector functions of CD8^+^ T cells (e.g., GZMB encoding granzyme B and IFNg encoding interferon-gamma), but also aberrantly regulates the maintenance of their stem-like properties and their transition toward a state of exhaustion. These two regulatory nodes act synergistically to severely impede the effective proliferation of CD8^+^ T cells and accelerate their functional exhaustion, thereby potently driving tumor immune evasion (81).

Furthermore, in CD4^+^ T cells, *in vitro* experiments have demonstrated that testosterone significantly inhibits the differentiation of murine CD4^+^ T cells toward the Th1 phenotype, while conversely promoting the secretion of immunosuppressive cytokines such as IL-10. The underlying molecular mechanism for this phenotypic shift lies in the fact that testosterone, via AR activation, upregulates the transcription of protein tyrosine phosphatase non-receptor type 1 (Ptpn1), which consequently blunts the pro-inflammatory signaling cascade induced by IL-12.Furthermore, AR signaling can suppress the activation n of the critical transcription factor STAT4, thereby further reducing IL-12 expression and launching a two-pronged assault that cripples the differentiation potential of Th1 cells (80, 82). This hormone-driven immune deviation is also corroborated in clinical settings: in transgender cohorts undergoing gender-affirming hormone therapy (GAHT), exogenous testosterone exhibits a pronounced propensity to inhibit the differentiation of CD4^+^ T cells into Th1 and Th17 phenotypes, while simultaneously skewing their differentiation toward immunosuppressive regulatory T cells (Tregs). These findings suggest that the direct remodeling of CD4^+^ T-cell differentiation trajectories by testosterone could provide critical insights for formulating sex-specific, personalized immunotherapy strategies in the future (83). Beyond its direct effects on T cells, androgens also induce immune evasion orchestrating the recruitment of immunosuppressive cellular networks. In hepatocellular carcinoma (HCC) models, androgens activate the NF-κB signaling pathway, enhancing the binding of the NF-κB complex to the promoter region of the CCL2 gene within tumor cells, thereby significantly upregulating the production of the chemokine CCL2. Subsequently, the overexpressed CCL2, operating through the CCL2-CCR2 chemotactic axis, potently drives the recruitment and intratumoral infiltration of peripheral Tregs. The massive infiltration of Tregs subsequently facilitates tumor immune evasion by directly suppressing the anti-tumor activity of γδ T cells (84). Within the innate immune compartment, AR signaling similarly acts as an “immune brake.” In macrophages, AR activation directly represses the transcription of the pro-inflammatory cytokine IL-1β, thereby fostering the formation of a local immunosuppressive microenvironment. Additionally, studies utilizing the macrophage-like THP-1 cell line have revealed that the AR signaling pathway can upregulate the expression of triggering receptor expressed on myeloid cells 1 (TREM1). This upregulation not only exacerbates immune tolerance within the microenvironment but also directly promotes the invasion and distant migration of cancer cells (85).

Estrogens exert their biological functions primarily through specific estrogen receptors (ERs). Cellular ERs are broadly classified into two major categories: classic nuclear estrogen receptors (nERs), which mainly include ERα and ERβ; and membrane-bound estrogen receptors, such as the G protein-coupled estrogen receptor (GPER, also known as GPR30) (86). Within the tumor microenvironment (TME), estrogen signaling frequently manifests a complex “double-edged sword” effect. On the one hand, clinical analyses of ER-positive metastatic breast cancer samples reveal a massive enrichment of CCL2- and SPP1-positive macrophages within the TME, displaying a profound immunosuppressive propensity. In contrast, primary tumor samples are predominantly enriched with FOLR2-and-CXCR3-positive macrophages, which possess anti-tumor potential (87). Furthermore, ERRα is deeply implicated in the metabolic reprogramming of tumor cells. Under hypoxic conditions within the TME, ERRα can downregulate NLRP3 expression to dampen inflammasome activity, thereby conferring apoptotic resistance to tumor cells. Concurrently, it accelerates glycolytic metabolism by activating rate-limiting enzymes of glycolysis, which induces pyroptosis resistance in endometrial cancer (ECs) and assists tumor survival in harsh milieus (88). On the other hand, extensive research validates that estrogens exert potent anti-tumor immune-activating effects across various non-reproductive system tumors. For instance, studies in female colorectal cancer (CRC) demonstrate that estrogens not only directly induce tumor cell apoptosis but also significantly upregulate the EOMES gene regulatory network in CD8^+^ T cells, thereby substantially boosting their immune-mediated cytotoxic functions. Simultaneously, the antigen-presenting capacity of B cells and their interactions with T-cell MHC-I molecules are markedly enhanced (89). The pivotal role of ERβ in anti-tumor immunity is equally undeniable. In ERβ-knockout murine models, the local infiltration of macrophages is aberrantly increased, whereas the infiltration of effector T cells and natural killer (NK) cells is severely compromised. Clinical observational data further corroborate that the mRNA expression levels of ERβ in tumor tissues are significantly and positively correlated with favorable patient prognosis (90). Intriguingly, animal experiments suggest that exogenous estrogens can actively remodel the immune function even in male primates. Following exogenous estrogen administration in male rhesus macaques, estrogens not only effectively suppressed endogenous testosterone secretion but also significantly enhanced systemic T-cell activation. This strongly implies that, in specific immune intervention therapies, supraphysiological levels of estradiol (E2) may harbor potential clinical utility as a novel immune-sensitizing agent (91).

In summary, estrogens and androgens exhibit highly complex and divergent activating or suppressive effects on the host immune system across distinct tumor microenvironments. Although this immune remodeling driven by endocrine networks is characterized by profound heterogeneity, deeply deciphering the core mechanisms of ER and AR signaling pathways in tumor immune evasion retains inestimable translational value. Moving forward, the strategic combination of targeted agents directed against these sex hormone receptor pathways (such as androgen deprivation therapy or selective estrogen receptor modulators) with immune checkpoint inhibitors will hold significant potential to provide a highly promising new avenue for overcoming immunotherapy resistance and realizing personalized precision medicine in oncology.

### 3.3 Metabolic reprogramming and sex differentiation in the tumor microenvironment

The aberrant metabolism of carbohydrates, amino acids, and lipids within the tumor microenvironment (TME) not only directly drives the malignant proliferation of tumor cells but also profoundly dictates the dynamic equilibrium of the immune ecology within the TME (92). Notably, this metabolic reprogramming exhibits pronounced sexual dimorphism. For instance, in lipid metabolism, studies have revealed that the levels of 25-hydroxycholesterol (25HC) display clear sex disparities, with its abundance being significantly higher in female mice than in males (93). Within the TME, the accumulation of lactate, the acidic milieu, and cytokines such as IL-4 and IL-13 synergistically upregulate the expression of cholesterol 25-hydroxylase (CH25H) in tumor-associated macrophages (TAMs). Interestingly, elevated levels of 25HC can actually restrain the immunosuppressive polarization of tumor-associated macrophages (TAMs). Consequently, the inherently higher abundance of 25HC in females helps mitigate TAM-mediated immunosuppression, thereby enhancing the intratumoral infiltration and preserving the cytotoxic activation of CD8^+^ T cells (94).

At the level of amino acid metabolism, sex-driven disparities similarly exert a critical regulatory role by disrupting histidine homeostasis. Clinical studies on hepatocellular carcinoma (HCC) have discovered that abnormally accumulated high levels of histidine in male patients upregulate the expression of the BUD23 gene, which concurrently downregulates multiple key immune signaling pathways, including MIF, CLEC, and MHC class II molecules. This metabolic aberration directly drives the polarization of macrophages toward an immunosuppressive subset and forces CD8^+^ T cells into a severe state of exhaustion, significantly impairing their activation and cytotoxic capacities. Further analysis based on the TCGA database indicates that silencing BUD23 can effectively reverse this metabolic defect and reinvigorate immune cell activity (95). Furthermore, epigenetic and metabolic crosstalk mediated by extracellular vesicles (EVs) is also subject to regulation by sex factors. For example, circPETH is a circular RNA transferred from TAMs to HCC cells via EVs. It not only accelerates glycolysis in HCC cells by promoting PKM2-catalyzed ALDOA-S36 phosphorylation but also increases the HuR-dependent stability of SLC43A2 mRNA. This leads to a massive hijacking of methionine and leucine by tumor cells in the TME, plunging cytotoxic CD8^+^ T cells into severe amino acid “metabolic starvation” and ultimately crippling anti-tumor immunity (96). Regarding the regulation of such non-coding RNAs (including miRNAs, lncRNAs, and circRNAs), Chen et al. proposed a hierarchical mechanistic framework: sex hormones, acting as the most upstream master regulators, systematically shape the local transcriptomic and epigenetic landscapes of the liver, thereby broadly influencing the transcription and expression of metabolism-related non-coding RNAs (97). In summary, while the aberrant metabolic networks in the TME remodel immune cell functions, their own evolutionary trajectories are profoundly governed by sex hormones, thereby imprinting a deep sex-specific signature on anti-tumor immunometabolism.

### 3.4 Sex-driven remodeling of the host gut microbiome and distal immune regulation

As a crucial bridge connecting the endocrine system with the systemic immune network, the composition and function of the host gut microbiome are profoundly regulated by sex hormones—a phenomenon widely conceptualized in the scientific community as the “Microgenderome.” In the context of remodeling the tumor immune microenvironment (TME), estrogens and androgens exhibit diametrically opposed evolutionary trajectories regarding the gut microbiome. Estrogens generally alleviate local intestinal inflammation, reverse systemic immunosuppression, enhance microbiome diversity and the richness of beneficial commensals, and effectively reduce the enrichment of opportunistic pathogens. Conversely, androgens tend to disrupt intestinal microbial homeostasis, exacerbate colonic inflammation and tumor growth, and ultimately undermine the clinical efficacy of tumor immunotherapies (98). For instance, studies have shown that estrogens can significantly reduce the Firmicutes/Bacteroidetes (F/B) ratio and optimize community diversity indices (Shannon and Simpson indices). In male mice supplemented with exogenous estrogens, this effectively increases the colonization abundance of commensal bacteria, thereby substantially mitigating their risk of developing colorectal cancer (99).

The distal regulation of tumor immunity by the gut microbiome primarily relies on its diverse repertoire of highly active metabolites, including tryptophan derivatives, primary/secondary bile acids, trimethylamine N-oxide (TMAO), and short-chain fatty acids (SCFAs). These microbiota-derived metabolites can enter the TME via the systemic circulation, systematically remodeling various immune and stromal components within it (100). Notably, the production trajectories of these critical metabolites also exhibit a high degree of sex specificity. For example, monocarboxylate transporter 1 (MCT1) in the intestinal epithelium regulates host glucose homeostasis in a strictly sex-dependent manner, and therapeutic intervention with estrogens can effectively counteract the glucose metabolic perturbations induced by MCT1 deficiency (101).

At the level of clinical anti-tumor therapy, the sexual dimorphism of the gut microbiome translates directly into heterogeneity in therapeutic responses. Metagenomic analyses have revealed that specific gut microbial communities enriched in female patients are highly correlated with enhanced estrogen signaling and more robust interferon (IFN)-mediated anti-tumor immune responses; whereas the specific taxa enriched in males exhibit diametrically opposite, immunosuppressive patterns. Furthermore, functional *in vitro* co-culture assays have corroborated that these sex-biased gut microbes can directly modulate the chemosensitivity of tumor cells (102).

Furthermore, it is imperative to distinguish biological sex from socio-cultural gender in this context. Beyond intrinsic hormonal regulation, gender-associated lifestyle disparities—such as distinct dietary preferences, alcohol consumption patterns, and occupational stress—can also profoundly and independently shape the gut microbiome composition, adding another layer of complexity to TME remodeling.

**Figure 2:**
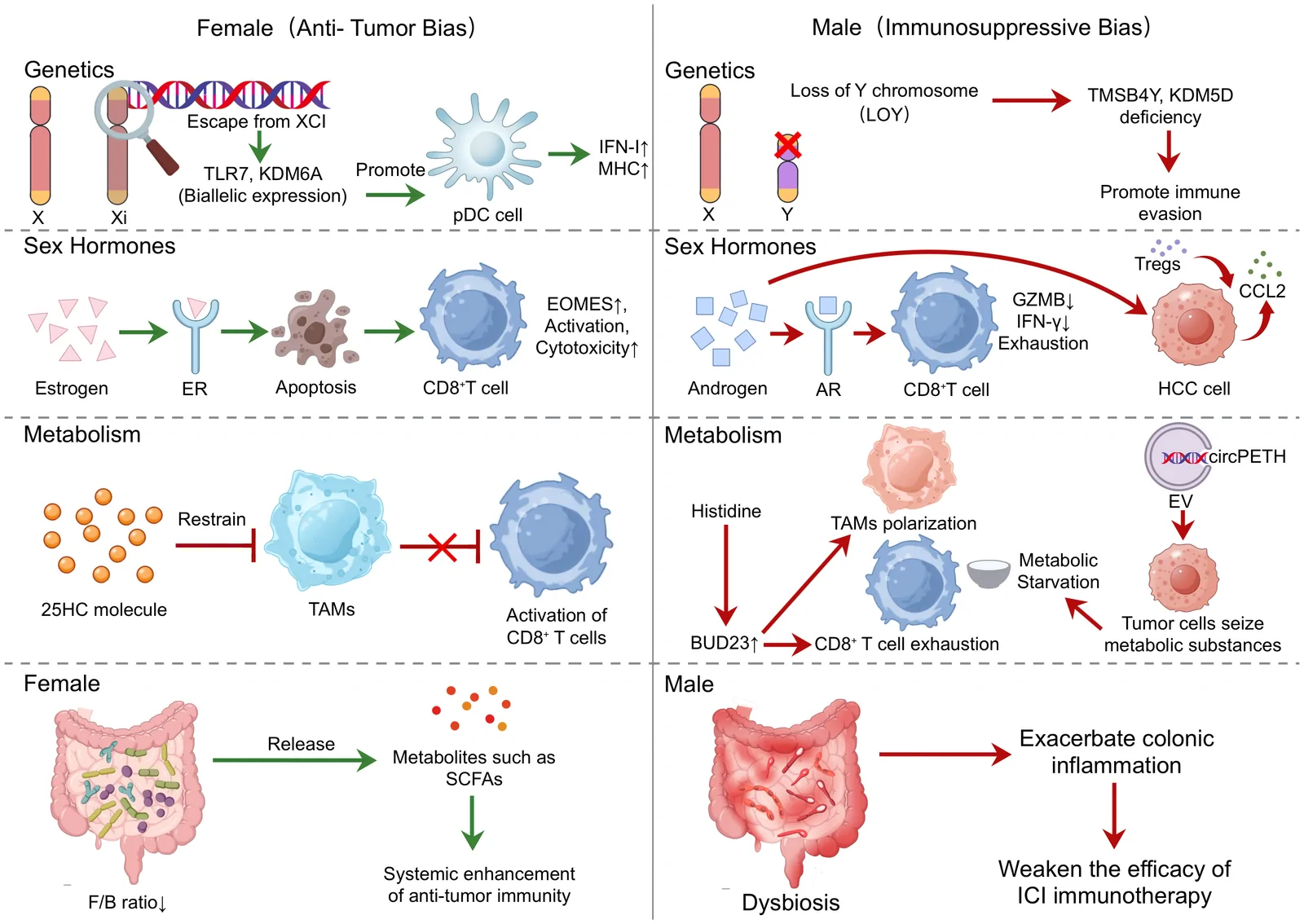
Sexual dimorphism in anti-tumor immunity orchestrated by four core mechanistic dimensions.

To provide a more intuitive illustration of the specific biological disparities across the aforementioned four core dimensions between sexes, and their ultimate impact on remodeling the anti-tumor immune microenvironment, we have systematically summarized these findings (see **Figure 2**; **Table 2**).

**Table 2:** Summary of the four core sex-disparate mechanisms driving anti-tumor immune heterogeneity.

Core regulatory dimension	Female	Male	References
Sex chromosome	X chromosome inactivation escape leads to the expression of both TLR7 and KDM6A alleles, enhancing IFN-I secretion and MHC expression.	Y chromosome deletion (LOY) leads to lose of TMSB4Y/KDM5D/UTY, promoting cell proliferation and immune evasion.	(65, 66, 70, 71, 73, 77, 78)
Sex hormone	Estrogens induce apoptosis, enhance CD8^+^ T cell function, and upregulate EOMES.	Androgens downregulate GZMB/IFNg, suppress Th1 cells, and promote Treg recruitment.	(81, 83, 84, 89)
Metabolic reprogramming	Higher levels of 25HC inhibit the immunosuppressive function of TAMs and enhance CD8^+^ cell activity.	Abnormal histidine metabolism leads to immune suppression of macrophages and depletion of CD8^+^ T cells.	(94, 95)
Intestinal microbiota	Reducing inflammation, increasing microbial diversity, and lowering the Firmicutes/Bacteroidetes (F/B) ratio.	Disrupting microbial balance, diminishing the efficacy of immunotherapy, and exacerbating colonic inflammation.	(98, 99)

## 4 Crosstalk between age and sex: reproductive senescence and endocrine interventions

### 4.1 Remodeling of the immune microenvironment by reproductive senescence-associated hormonal fluctuations and HRT

Drastic hormonal fluctuations, particularly during female menopause and the male climacteric, exert profound impacts on the host immune landscape and the gut microbiome. For example, in postmenopausal women, the precipitous drop in systemic estrogen levels leads to a relatively androgen-excessive state (103). Notably, following reproductive senescence and the abrupt decline in estrogen signaling, the female-biased innate anti-tumor immune advantages—previously conferred by the synergy of X-chromosome inactivation (XCI) escape and estrogens—become significantly attenuated. This abrupt endocrine shift heavily remodels the tumor immune microenvironment (TME) and influences therapeutic responses. Taking triple-negative breast cancer (TNBC) as an example, premenopausal patients generally exhibit better prognoses than postmenopausal patients. Gene pathway analyses reveal that the premenopausal state upregulates immune-related processes—such as MHC-mediated antigen presentation, T-cell activation and differentiation, and type II interferon signaling—while downregulating oncogenic PI3K-AKT and MAPK pathways. At the cellular level, premenopausal tumors show higher infiltration of CD8^+^ T cells, mature DCs, and macrophages (104). Similar distinct TME disparities between pre- and postmenopausal states have also been observed in high-grade serous ovarian cancer (HGSOC) (105).

Furthermore, menopausal estrogen depletion severely remodels the gut microbiome, causing a Firmicutes/Bacteroidetes (F/B) imbalance and a substantial decrease in short-chain fatty acid (SCFA) production (99). The dual hit of advancing age and estrogen decline significantly reduces the abundance of beneficial bacteria while increasing opportunistic pathogens (29, 98), thereby impairing intestinal barrier and immune defense functions, and exacerbating systemic inflammation (106). Similarly, the decline in testosterone levels during the male climacteric (andropause) triggers gut dysbiosis and elevated pro-inflammatory cytokines (107–109). Against this backdrop, Hormone Replacement Therapy (HRT) emerges as a crucial intervention for postmenopausal patients, demonstrating immense translational potential in remodeling the immune composition. Emerging evidence suggests that HRT can partially reverse the systemic immunosuppression induced by reproductive senescence by replenishing estrogenic signals. It not only helps restore the homeostasis of peripheral immune cells but also reinvigorates effector T-cell functions within the TME, thereby holding promise for augmenting the efficacy of immunotherapies. However, exogenous hormonal interventions are highly complex; for instance, the inappropriate use of estrogen-progestin replacement therapy may paradoxically increase the risk of meningioma in menopausal women (110). Therefore, in personalized immuno-oncology, meticulously weighing the immunological benefits of HRT against its potential risks is of critical importance.

### 4.2 Genomic deletions and personalized immuno-endocrine therapies

At the genomic level, the loss of Y chromosome (LOY) is closely associated with aging and serves as a major driver of increased cancer incidence in males. Analyses of peripheral blood from male patients with prostate, pancreatic, and colorectal cancers reveal that the proportion of LOY can serve as a crucial predictive biomarker for carcinogenesis and immune evasion (111, 112).

Interestingly, from a clinical outcome perspective, these profound sex-driven mechanistic differences translate into distinct therapeutic efficacies. Extensive meta-analyses of clinical trials, such as those conducted by Conforti et al., robustly indicate that male patients often derive a significantly greater survival benefit from immune checkpoint inhibitors (ICIs) compared to females (113). Mechanistically, while females generally mount stronger baseline immune responses, their tumors often adapt by developing a highly immunosuppressive TME, characterized by a greater abundance of regulatory T cells (Tregs), MDSCs, and higher expression of inhibitory checkpoints. Conversely, male tumors frequently exhibit a T-cell-excluded phenotype and impaired neoantigen presentation (114). This paradoxical phenomenon suggests that releasing the severe, androgen- and LOY-driven “immune brakes” in males may yield a more dramatic therapeutic rebound than attempting to overcome the complex, multi-layered immunosuppressive networks evolved in female patients.

Regarding endocrine interventions, sex hormones are considered among the most critical sex-related factors influencing immunotherapy efficacy (81). Estrogens can shape an immunosuppressive local TME, resulting in suboptimal responses to ICIs in the majority of patients with estrogen receptor (ER)-positive breast cancer. Preclinical models confirm that combining chemotherapy with fulvestrant (an ER downregulator) can effectively enhance anti-tumor immune responses and increase ICI sensitivity, even when tumor growth is no longer strictly estrogen-dependent (115). The dynamic fluctuations in estrogen levels profoundly affect therapeutic outcomes in sex-related cancers. For instance, in patients with early-stage ER-positive breast cancer, 5 years of extended adjuvant therapy with aromatase inhibitors (which block androgen-to-estrogen conversion) significantly reduces the distant recurrence rate by approximately 25% (116). For premenopausal women, an estrogen-sensitizing pathway comprising E-cadherin, β-catenin, ER-α, and GRPR promotes tumor development; developing selective GRPR antagonists and YAP1-targeted drugs holds promise for improving treatment outcomes in young female patients (117). Additionally, fibroblasts in the ovaries of premenopausal gastric cancer patients highly express ER; subsequent estrogen stimulation activates the MDK (midkine)-LRP1 signaling axis, thereby driving the ovarian metastasis of gastric cancer (118).

For prostate cancer, a highly prevalent male malignancy heavily dependent on androgen receptor (AR) signaling, androgen deprivation therapies (ADTs) and anti-androgens are classic treatments. Interestingly, when male patients and their tumor cells express ERα, selective estrogen receptor modulators (SERMs) can serve as effective therapeutics against such prostate tumors (119). Studies on ADT in elderly men with prostate cancer have demonstrated that transdermal estradiol (tE2) supplementation not only further suppresses testosterone levels but also effectively mitigates the estrogen-deficiency side effects induced by LHRH agonists, while simultaneously circumventing the thromboembolic risks associated with oral estrogens (120). In hepatocellular carcinoma (HCC), estrogens can further mediate sex disparities in tumor development by activating the LCAT/HDL-C axis to reduce cholesterol biosynthesis, providing novel endocrine intervention strategies for the prevention, prognostication, and personalized treatment of liver cancer (121).

### 4.3 Summary of age-sex crosstalk

In summary, the crosstalk between age and sex is most prominently manifested during reproductive senescence. The precipitous decline in sex hormones during menopause or andropause, coupled with age-related genomic alterations such as LOY, synergistically exacerbates immunosenescence and TME deterioration. However, this highly dynamic intersection also provides a crucial window of therapeutic opportunity for personalized medicine. Precision endocrine interventions—such as HRT, SERMs, or ADTs—hold immense potential to not only reverse age-driven immunosuppression but also synergize with immune checkpoint inhibitors (ICIs), thereby providing a powerful therapeutic armamentarium for truly personalized immuno-oncology.

## 5 Conclusion and future perspectives

In summary, the remodeling of the tumor immune microenvironment (TME) is not solely dictated by the genetic mutations of the tumor cells themselves; rather, fundamental host variables—namely, age and physiological sex—play a critical “steering” role in this process. This review has systematically delineated the core driving mechanisms across these two dimensions. At the level of age, the progressive functional decline of T/B lymphocytes and innate immune subsets, driven by immunosenescence and intertwined with the chronic sterile inflammatory networks fueled by inflammaging (SASP), ultimately constructs a robust immunosuppressive microenvironment in the aging host. At the level of sex, the genetic imprints of sex chromosomes (e.g., escape from X-chromosome inactivation and loss of Y chromosome), transcriptional regulation mediated by sex hormone receptors, and the sex-biased nature of metabolic networks and the gut microbiome collectively shape the “dimorphism” in anti-tumor immune phenotypes between males and females.

A profound understanding of the immunological effects of age and sex bears tremendous translational value for breaking through current bottlenecks in cancer immunotherapy. These two core variables not only explain why identical immune checkpoint inhibitors (ICIs) elicit significantly different survival benefits and immune-related adverse events (irAEs) across distinct patient cohorts, but also expose the profound limitations of conventional “one-size-fits-all” immunotherapeutic strategies. For instance, fimmu.2026.1918543elderly patients, burdened by overall immune deterioration and impaired antigen recognition, face heightened risks of primary resistance and toxicity challenges. Conversely, although male patients suffer from severe, androgen-driven T-cell exhaustion, they may paradoxically unleash more pronounced anti-tumor efficacy once the “immune brakes” are released by ICIs.

Looking to the future, realizing true “precision cancer immunotherapy” urgently necessitates a paradigm shift in both basic research and clinical practice. First, in preclinical animal studies, the traditional reliance on single-sex models (typically young females) must be thoroughly abandoned. It is imperative to strictly incorporate “Sex as a Biological Variable (SABV)” into experimental designs and widely adopt natural aging models to authentically recapitulate the complex immune landscape of clinical patients. Second, future clinical trial designs and data reporting must mandate granular, stratified statistical analyses based on age and sex, thereby uncovering differentiated predictive biomarkers for efficacy. Finally, the development of novel combination therapy strategies rooted in age- or sex-specific mechanisms—such as combining ICIs with senolytics targeting senescent cells, endocrine interventions (e.g., androgen deprivation therapy or selective estrogen receptor modulators [SERMs]), or sex-specific gut microbiome modulators—will hold significant potential to pave the breakthrough path for the next generation of personalized immuno-oncology.
