# Correction: Age and sex: dual drivers remodeling the anti-tumor immune microenvironment and shaping personalized immuno-oncology

**DOI:** 10.3389/fimmu.2026.2027356

**Published:** 2026-10-26

**Authors:** Yipeng Wang, Tianshu Wang, Ying Xi, Yihan Wang, Yibo Bai, Tao Zhang

**Affiliations:** 1 Department of Gastric Surgery, Cancer Hospital of Dalian University of Technology, Liaoning Cancer Hospital & Institute, Shenyang, Liaoning, China; 2 Medical Oncology Department of Gastrointestinal Tumors, Cancer Hospital of Dalian University of Technology, Liaoning Cancer Hospital & Institute, Shenyang, Liaoning, China; 3 Department of Laboratory Medicine, Shengjing Hospital of China Medical University, Shenyang, Liaoning, China; 4 Liaoning Clinical Research Center for Laboratory Medicine, Shenyang, Liaoning, China; 5 China Medical University, Shenyang, Liaoning, China

**Keywords:** anti-tumor immunity, immune evasion, immunosenescence, sex differences, tumor microenvironment

In the published article, there was an error in Affiliation 1 and Affiliation 2.

Affiliation 1:

Original:

“1 Department of Gastric Surgery, Dalian University of Technology Affiliated Tumor Hospital, Liaoning Cancer Hospital, Liaoning Provincial Tumor Research Institute, Shenyang, Liaoning, China”

Corrected:

“1 Department of Gastric Surgery, Cancer Hospital of Dalian University of Technology, Liaoning Cancer Hospital & Institute, Shenyang, Liaoning, China”

Affiliation 2:

Original:

“2 Medical Oncology Department of Gastrointestinal Tumors, Dalian University of Technology Affiliated Tumor Hospital, Liaoning Cancer Hospital, Liaoning Provincial Tumor Research Institute, Shenyang, Liaoning, China”

Corrected:

“2 Medical Oncology Department of Gastrointestinal Tumors, Cancer Hospital of Dalian University of Technology, Liaoning Cancer Hospital & Institute, Shenyang, Liaoning, China”

The authors apologize for this error and state that this does not change the scientific conclusions of the article in any way.

The original article has been updated.

